# Prediction of Solitary Plasmacytoma of Bone in Elderly Patients: A Nomogram and a Risk Classification System for Overall Survival

**DOI:** 10.1155/2022/7387416

**Published:** 2022-06-01

**Authors:** Xi Zhang, Jiahao Yang, Zhuanhua Tian, YOUSSEF KHABIR, Dan Wang

**Affiliations:** Department of Orthopaedic Surgery, The First Affiliated Hospital of Zhengzhou University, Zhengzhou, Henan, China

## Abstract

**Background:**

Solitary plasmacytoma of bone (SPB) is an isolated plasmacytoma of bone origin, most commonly seen in the elderly, with a poor prognosis. So far, there is no precise nomogram to predict the overall survival (OS) of elderly patients with SPB. Our goal is to construct and validate a nomogram for elderly patients with SPB.

**Methods:**

This study collected all elderly patients with SPB in the Surveillance, Epidemiology and End Results (SEER) database from 2000 to 2018, and the variables included were age, sex, race, marital status, primary site, grade, stage, surgery, chemotherapy, and radiotherapy. Independent prognostic factors were identified using univariate and multivariate Cox analysis. The nomogram was constructed to predict 1-, 2-, and 3-year OS of elderly patients with SPB. The receiver-operating characteristic (ROC) and the calibration curves were used to differentiate and calibrate the nomogram. The clinical validity of the nomogram was evaluated by decision curve analysis (DCA). The total OS scores of all elderly SPB patients were calculated and divided into two risk subgroups for comparison.

**Results:**

A total of 1837 patients diagnosed with SPB were screened from the SEER database, with a final inclusion of 1180 patients (age ≥ 60 years). Age, radiotherapy, and marital status were significantly correlated with OS. These characteristics were further incorporated into the creation of the nomogram for predicting 1-, 2-, and 3-year OS of elderly patients with SPB. For this predictive model, the area under the ROC curves, calibration curves, and DCA have good performance in terms of differentiation, consistency, and validity, respectively. In addition, patients in the high-risk group (≥96) had a worse prognosis than those in the low-risk group (<96).

**Conclusion:**

We constructed a nomogram and a risk classification system that could provide an intuitive and effective tool for clinicians to better predict the OS of elderly SPB patients.

## 1. Introduction

Plasmacytoma is a primary and systemic malignancy originating from the bone marrow and characterized by clonal proliferation of plasma cells. Plasmacytomas include extramedullary plasmacytoma (EMP), solitary plasmacytoma of bone (SBP), and multiple myeloma (MM) [1]. SBP is a rare disease with a high recurrence rate, a cumulative incidence of 0.15/100,000, and a poor prognosis for patients with SPB over 60 years of age [2, 3]. The diagnosis of SPB is currently based mainly on histological examination and confirmed by tissue biopsy and radiology [4, 5]. Patients with SPB may exhibit neurological impairment or severe pain because of spinal instability or pathological fractures [6, 7]. In addition, these performances are usually used as primary symptoms along with a poor prognosis in the elderly population. For both surgeons and physicians, it is necessary to pay attention to these patients in our clinical work. Radiotherapy is the standard of care for SPB, even in patients who have undergone complete tumor resection. Although radiotherapy has excellent local control rates, SPB has a high recurrence rate, with approximately 2/3 of patients in advanced stages progressing to additional solitary or multiple plasmacytomas [8].

Several studies [8, 9] have investigated the potential risk factors for SPB. However, they have only analyzed relevant prognostic factors, and the data included were not comprehensive, such as the lack of data on radiotherapy or chemotherapy, which did not accurately predict the prognosis of patients with SPB. Furthermore, these studies have not limited the research population to the vulnerable elderly population and have not provided a good visualization model. For example, nomograms have been widely used in clinical predictions, which can not only help patients to assess their risks of disease but also guide doctors to make proper medical decisions [10]. So, we analyzed the SEER database, which collects data from cancer registries in 18 different regions, accounting for about 28% of the United States population [11]. The objective of this study was not only to identify independent factors affecting survival outcomes in elderly patients with SPB but also to provide a nomogram to accurately predict the probability of survival in elderly patients, which may be of interest.

## 2. Materials and Methods

### 2.1. Study Population and Data Collection

**Figure 1:**
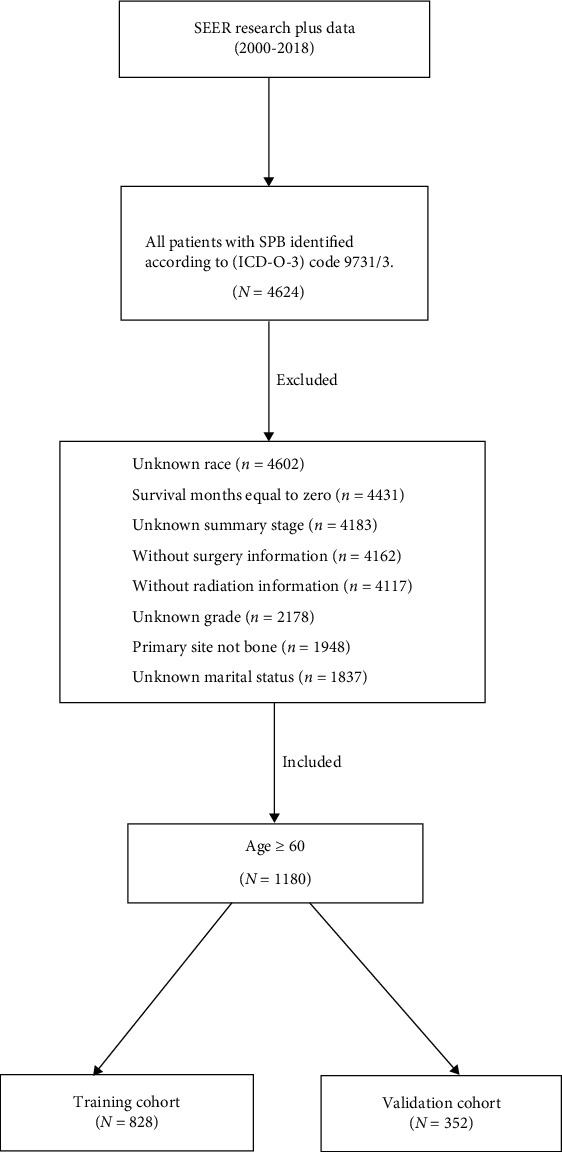
The study flow diagram of the selection process.

We screened all cases of SPB from the SEER database using the International Classification of Diseases for Oncology (ICD-O-3) code 9731/3. Cases with peripheral blood, bone marrow, or other extramedullary organ involvement and those with incomplete information were excluded from this study. The flow chart for data selection is detailed in Figure 1. The following relevant data were extracted: age (≥60 years old), sex (female or male), race (black, white, others), marital status (no, yes), primary site (trunk, extremities, facial/skull bone), grade (pre-B; grade I, good differentiation; grade II, moderate differentiation; grade III, poor differentiation; grade IV, no differentiation), stage (localized, distant), surgery (no, yes), chemotherapy (no, yes), and radiotherapy (no, yes). Due to the retrospective nature of the study and the absence of personally identifiable information, we did not require review board approval or informed consent from patients.

### 2.2. Statistical Analysis

We collected 1180 cases and randomly divided them into training cohorts (70%) and validation cohorts (30%). The patient's optimal cut-off value was obtained by X-tile software (Yale University, USA). Independent prognostic factors were identified using univariate and multivariate Cox analysis. The nomogram was constructed to predict 1-, 2-, and 3-year OS of elderly patients with SPB. We constructed receiver-operating characteristic (ROC) curves and calibration curves to verify the differentiation and calibration of the nomogram and evaluated the validity of the model using decision curve analysis (DCA) [12, 13]. In addition, we calculated the total scores of all patients based on the nomogram and used X-tile software to find the optimal cut-off value for the total scores, subsequently dividing the patients into two risk subgroups. Furthermore, survival curves for all variables were constructed using the Kaplan-Meier method to facilitate the analysis of survival trends. Graphic production and statistical analysis were performed using R software, and bilateral *p* values < 0.05 were considered to be statistically significant.

## 3. Results

### 3.1. The Characteristics of the Study Population

Based on the inclusion and exclusion criteria, we ultimately included 1837 patients with SPB from the 2000-2018 SEER database, of which a total of 1180 were aged ≥60 years. The training cohort and the validation cohort consisted of 828 and 352 individuals, respectively, for the construction of the nomogram and validation. Patients were predominantly male (60.9% and 58.8% for the training and validation cohorts, respectively) and white (79.2 and 85.5%), had a marital status of yes (67.3 and 63.1%), had undergone radiotherapy (77.4 and 75.3%), and localized (82.5 and 82.7%). Concerning the pathologic grades and primary site, pre-B cell (96.9 and 98.0%) and trunk (75.4 and 80.7%) were the most common.  Table 1 shows in detail the demographic characteristics of these patients.

### 3.2. Prognostic Factors in Elderly Patients with SPB

**Figure 2:**
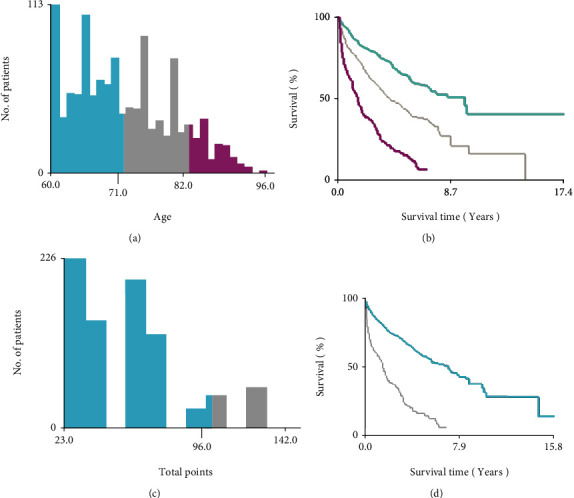
Identification of optimal cut-off values of age and risk scores by X-tile analysis. Optimal cut-off values of age were identified as 71 and 82 years based on overall survival (a). Optimal cut-off values of risk scores were identified as 96 scores based on overall survival (c). Histogram and Kaplan–Meier analyses were developed based on these cut-off values (b, d).

**Figure 3:**
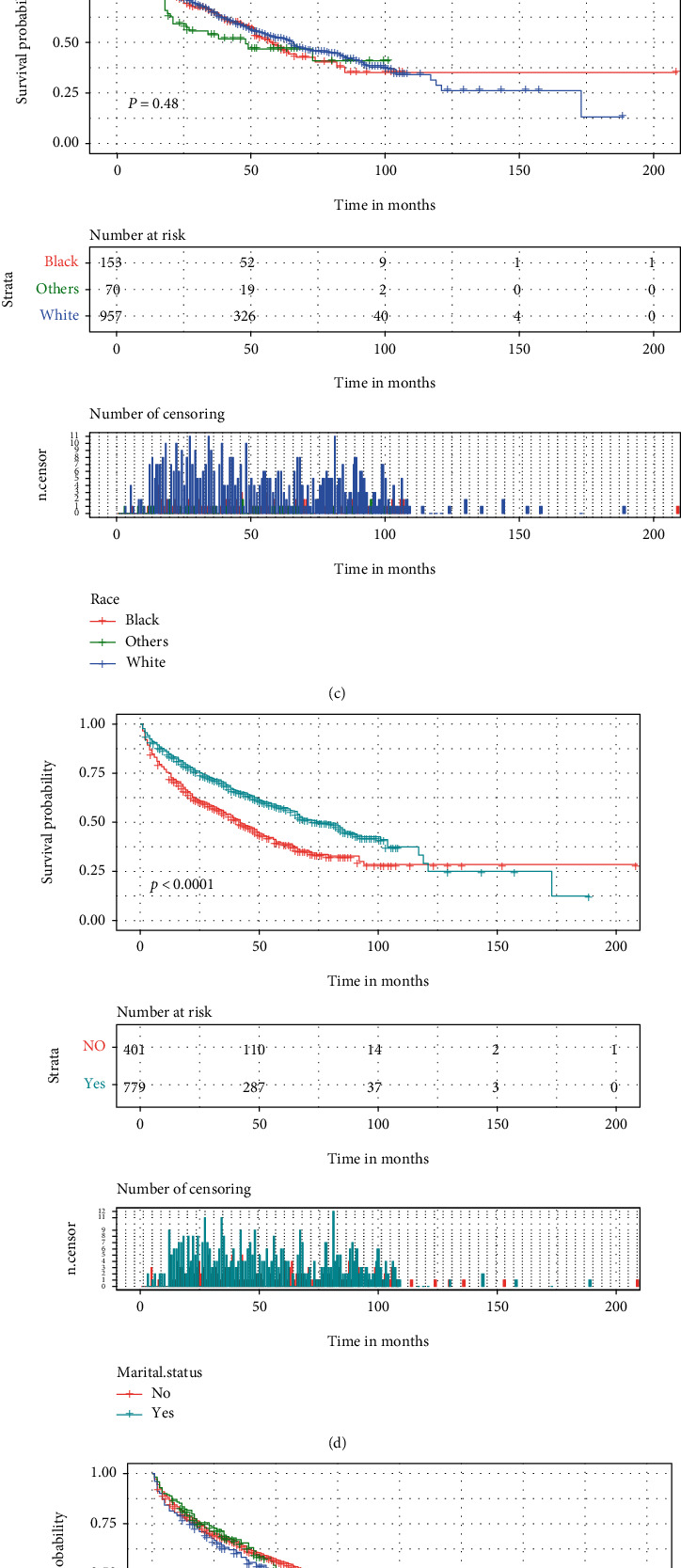
Kaplan–Meier curves of variables were performed for OS in elderly patients with SPB. (a) Age, (b) sex, (c) race, (d) marital status, (e) primary site, and (f) grade.

**Figure 4:**
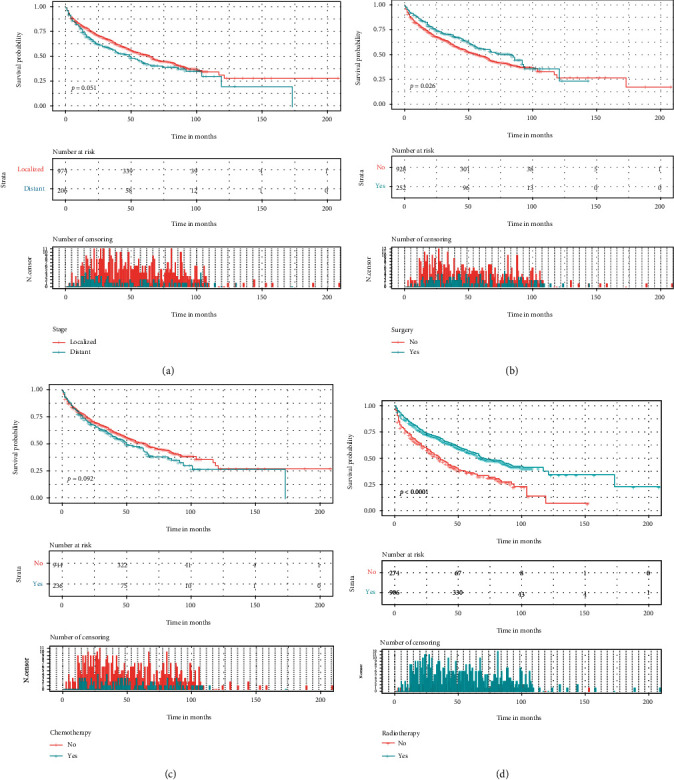
Kaplan–Meier curves of variables were performed for OS elderly patients with SPB. (a) Stage, (b) surgery, (c) chemotherapy, and (d) radiotherapy.

The optimal cut-off values of the age through the X-tile software were 71, 82, years (Figure 2). Univariate and multivariate analyses of OS in elderly patients with SPB were conducted as shown in Table 2. From the Cox univariate analysis, we derived that patients' age (*p* ≤ 0.001), marital status (*p* ≤ 0.001), and radiotherapy (*p* ≤ 0.001) were significantly associated with OS, while patients sex, race, primary site, stage, grade, surgery, and chemotherapy were not significantly correlated with OS. Because the *p* values of surgery in the cox univariate and log-rank analyses were 0.083 and 0.026, respectively, and because of the importance of surgery in clinical care, we also included surgery in the Cox multivariate regression analysis to avoid missing variables. The Cox multifactor regression analysis further showed that age (71-82 years old, HR = 1.856, 95%CI = 1.469–2.345, *p* ≤ 0.001; >82 years old, HR = 4.305, 95%CI = 3.256–5.692, *p* ≤ 0.001), marital status (yes, HR = 0.670, 95%CI = 0.544–0.824, *p* ≤ 0.001), and radiotherapy (yes, HR = 0.695, 95%CI = 0.557–0.868, *p* ≤ 0.001) were independent prognostic factors for OS in elderly patients with SPB. The Kaplan-Meier curves of all variable factors in OS were drawn (Figures 3 and 4).

Construction and validation of a nomogram for elderly patients with SPB.

**Figure 5:**
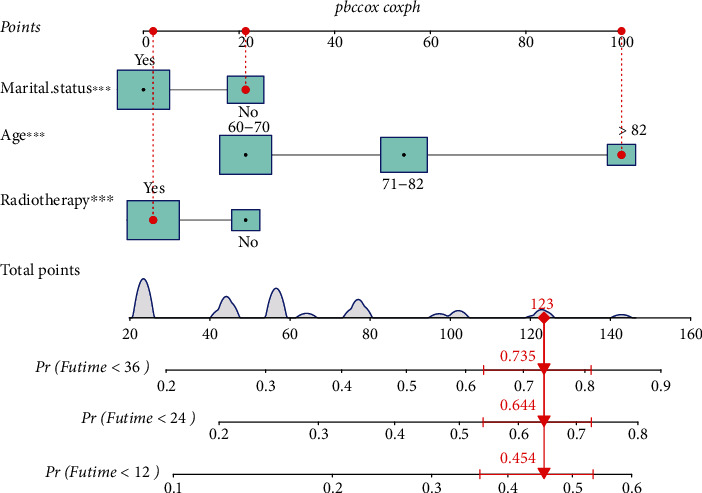
Nomogram to predict overall survival (OS) in elderly patients with SPB.

**Figure 6:**
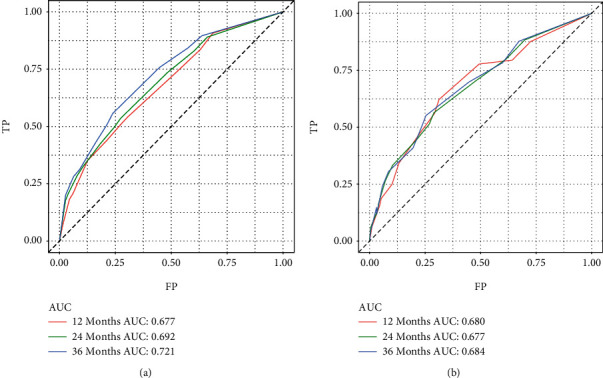
Receiver-operating characteristic curves of 1, 2, and 3 years in the training cohort (a) and validation cohort (b).

**Figure 7:**
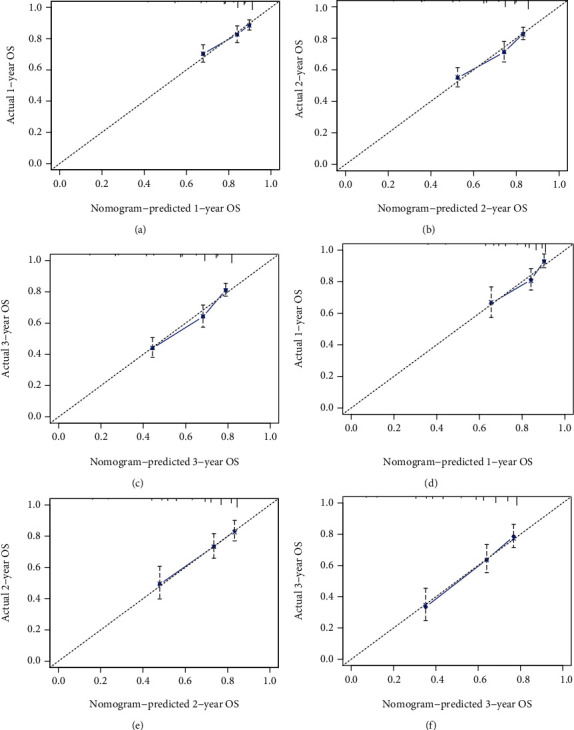
Calibration plots of the nomogram for 1-, 2-, and 3-year predicting overall survival (OS) in elderly patients with SPB of the training cohort (a–c) and validation cohort (d–f).

**Figure 8:**
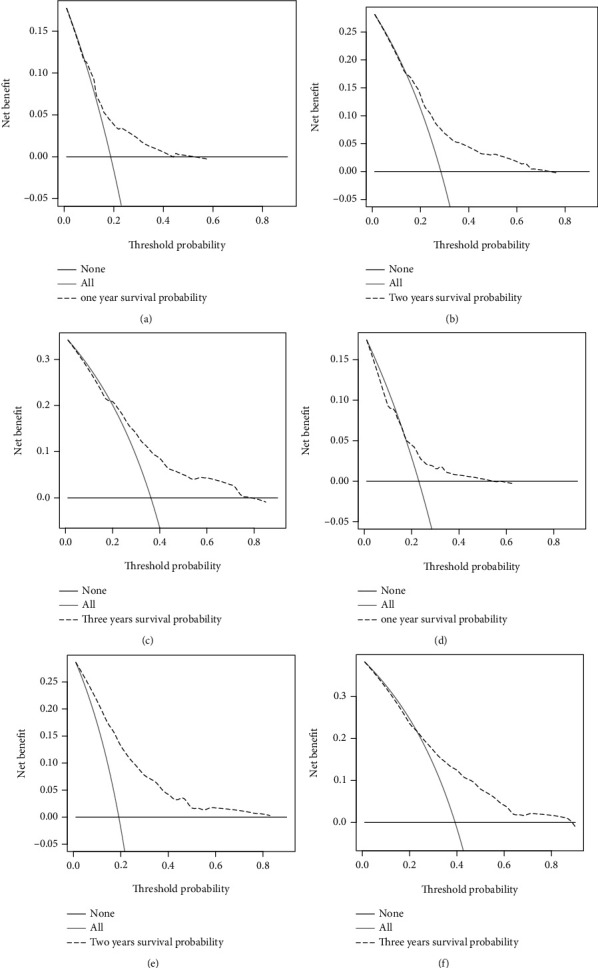
Decision curve analysis of the nomogram for 1-, 2-, and 3-year predicting overall survival (OS) in elderly patients with SPB of the training cohort (a–c) and validation cohort (d–f).

According to the independent prognostic factors screened by the above analysis, we constructed a nomogram based on R language to predict the prognosis of elderly patients with SPB (Figure 5). The area under the ROC curve (AUC) values of 12-, 24-, and 36-month OS in the training (Figure 6(a)) and validation cohorts (Figure 6(b)) were 0.677, 0.692, and 0.721 and 0.680, 0.677, and 0.684, respectively, displaying excellent predictive differentiation. And the calibration curve for both the training and validation cohorts showed excellent consistency as well (Figure 7). We plotted the DCA curves for the training and validation cohorts at 1, 2, and 3 years, showing the best net benefit of the nomogram and demonstrating the advantages of the nomogram (Figure 8).

### 3.3. Risk Classification System

**Figure 9:**
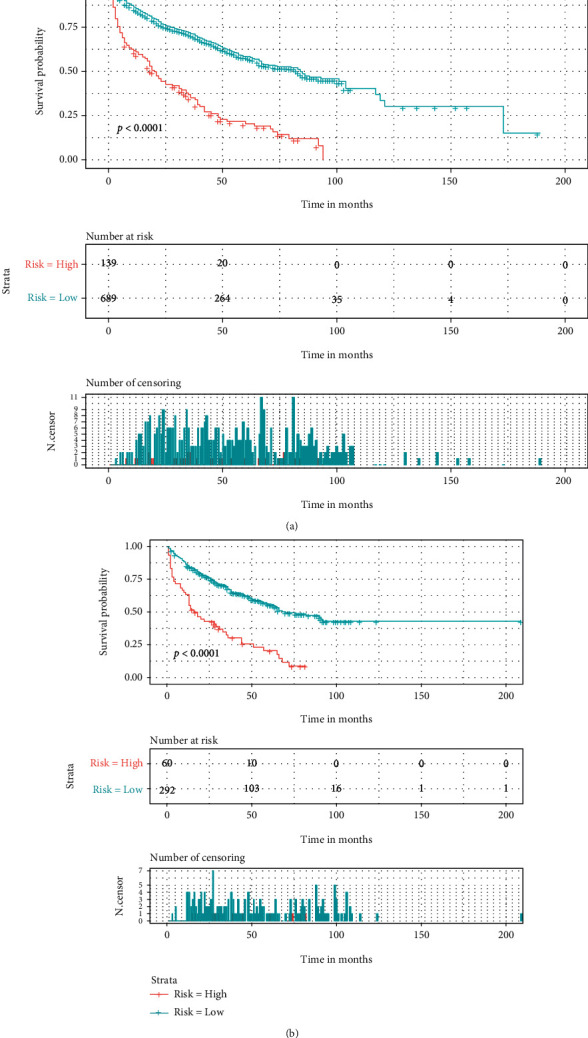
Kaplan–Meier survival analysis for both the training cohort (a) and the validation cohort (b).

In addition, we calculated the total scores of all elderly patients with SPB based on the nomogram and used the X-tile software to determine that a score of 96 was the optimal cutoff value for OS. And we attempted to establish a risk classification system for this nomogram and divided the patients into two risk subgroups of high risk (≥96) and low-risk (<96) by the X-tile software. Survival curves for both the training and validation groups showed good prognostic stratification, with the high-risk group having a worse prognosis than the low-risk group (Figure 9).

## 4. Discussion

Solitary plasmacytoma of bone is a type of malignant plasmacytoma disease with a rare nature and a relatively high recurrence rate [14, 15]. Moreover, SPB often occurs in the elderly [16]. To better help patients and physicians predict the probability of survival after the disease, it is urgent to establish a predictive model to give the correct guidance. The nomogram graphically quantifies the predictive model as a numerical estimate of the probability of survival, which is tailored to the characteristics of individual patients. Nomograms that were proved convenient and effective have been constructed for a variety of cancers [17–19]. To the best of our knowledge, this study is the first to develop and validate an OS nomogram for elderly patients with SPB. Based on the 1180 cases extracted from the SEER database, we developed a comprehensive predictive model for 1-, 2-, and 3-year OS in elderly patients with SPB.

In this study, we found that age, marital status, and radiotherapy were independent prognostic factors for OS in elderly patients with SPB, and we further developed a nomogram to predict survival outcomes, which showed good predictive performance for both internal and external validation. As shown in the red line marked in Figure 5, we had assumed an elderly patient of SPB who is more than 82 years old, living alone, and has taken radiotherapy, we could draw a total score of 123 points and the mortality of 3-year, 2-year, and 1-year were 73.5%, 64.4%, and 45.4%, respectively.

We found a significant difference in survival trends among patients aged 60-70, 71-82, and >82 years on the Kaplan-Meier curve (*p* < 0.0001), with an increasingly worse prognosis with increasing age. Many studies [20–22] have shown that age is associated with survival outcomes in a variety of cancers. In addition, according to the results of previous retrospective studies by Knobel et al. [23] and Jawad and Scully [24], age (>60 years) was a poor prognostic factor for plasmacytoma in both univariate and multifactorial analyses. This is in keeping with our findings. The incidence of SPB in men was higher than in women (1.5 : 1), but there was no significant difference in prognosis between women and men, which was inconsistent with the study by Ramsingh et al. [25], which showed that women had a poorer prognosis. As shown in Table 1, whites have a higher incidence (79.2%), and according to Ramsingh et al. [25], blacks with solitary plasma cell tumors have significantly worse survival rates than other ethnic groups. In contrast, in our study, the race had no significant effect on the outcome of patients with SPB. The reason for the discrepancy between the two studies mentioned above is considered to be caused by the inconsistency of the study population; the study by Ramsingh et al. selected all SP patients diagnosed from 1988 to 2004, whereas our study included elderly SPB patients diagnosed from 2000 to 2018.

Marital status, as a sociodemographic factor, has been well documented as an independent prognostic factor for multiple cancer types [26–28]. Marital status included married, divorced, separated, widowed, and single, and patients with SPB who have a partner have better survival. We consider it from two aspects: on the one hand, partners urge patients to screen for health conditions and advocate for aggressive treatment [29, 30]; on the other hand, marital status provides hope for patients. Evidence has also found that married cancer patients are diagnosed with cancer earlier than unmarried patients [27]. Doctors should take into account the marital status of the patients when calculating the probability of survival for SPB patients, and for those elderly who are without a partner, we may want to inform the patient's family or caregiver to increase care and attention. The effect of lesion location on prognosis in patients with SPB remains unclear. The lesion was more common in the trunk bone (75.4%), which was consistent with most previous reports. And the survival of patients with SPB of the trunk bone was similar to that of patients with SPB of other parts of the body [2, 8]. In our study, we found an interesting phenomenon that the survival of patients with SPB of the head and face was poorer, although the survival differences were not significant. The causes of this phenomenon are not clear, but one possible explanation is that patients with SPB are relatively rare in the skull and facial bones, and clinicians are prone to misdiagnosis or missed diagnosis, delaying the optimal time for efficacy. In addition, It was reported that anaplastic (grade IV) plasmacytoma and aggressive B-cell lymphoma had some common pathologic features, poor prognosis, and possible progression to Epstein-Barr virus infection and immunosuppression [31]. This could explain why SPB has a lower survival rate due to the poor differentiation.

Solitary plasmacytoma of bone is highly sensitive to radiation, and radiotherapy has become the first-line treatment for most patients at doses of up to 40-50 Gy [32], with excellent local control rates. However, whether surgery and chemotherapy play an active role in treatment is a long-discussed topic. In previous studies [4, 23, 33], radiotherapy alone as the only treatment option is more effective than both chemotherapy and surgery. Our study showed that SPB patients treated with radiotherapy had higher overall survival in both univariate and multivariate analyses (Figure 4 and Table 2). However, our study also found that surgery and chemotherapy were not independent prognostic factors for elderly patients with SPB. Surgery is meaningful only in log-rank univariate analysis. Shen et al. [8] concluded that surgery plays a vital role in the prognosis of patients with SPB, and Xie et al. [9] found that surgery might delay the progression of MM in younger patients with SBP, who may benefit more from surgical treatment. In this study, we believed that surgery did not increase survival in elderly patients with SPB. Currently, the consensus among surgeons is that once extremity or vertebral bone destruction occurs, patients with SBP may experience pathological fractures, extremity instability, vertebral collapse, spinal cord or nerve root compression, and spinal instability, and early surgery is recommended for these cases. The objective of early surgery is to prevent compression of the spinal cord or nerve roots, to remove the tumor lesion, and to reestablish stability of the extremities and spine. Therefore, in elderly patients with SPB, we should perform palliative surgery before radiotherapy [9] to improve the patient's quality of life rather than overall survival. There is evidence that adjuvant chemotherapy can reduce the risk of progression of SPB to MM [34]. However, the efficacy of chemotherapy on SPB is still inconclusive. A prospective study that evaluated the efficacy of chemotherapy in combination with radiation therapy in 53 patients has concluded that combination therapy is beneficial for patient survival and disease progression [35]. Recently Kumar et al. [36] showed that myeloma is highly correlated with angiogenesis and reported that targeted agents such as thalidomide are a new approach to treat SPB. From our Kaplan-Meier curves, patients using chemotherapy instead had poorer survival, and we considered that the chemotherapy in these patients was administered after progression to MM, which contributed to this result. Therefore, we should think more about the individual application and the research and invention of new drugs in the future. In conclusion, the correct application for radiotherapy, surgery, and chemotherapy needs to be validated in future prospective studies. In addition, we divided the predictive model into high and low risk using a 96-point cut-off, which, combined with the survival curve plots, allowed us to well assess and interpret the survival prognosis of elderly patients with SPB. Moreover, patients can also clearly see their survival trend based on their total scores.

There are several limitations to this study. First, as a retrospective study, it inevitably led to a degree of bias that needs to be further validated in subsequent prospective studies. Second, we used the same central database for both internal and external validation, and it would have been preferable to validate the nomogram using data from multiple centers to increase its reliability. Third, considering the lack of laboratory tests, gene expression items, radiotherapy doses, and surgical methods in the SEER database, future studies should attempt to include these factors to create a more effective nomogram of SPB in the elderly.

## 5. Conclusions

Age, radiotherapy, and marital status were identified as independent prognostic factors for elderly SPB patients, and surgery was excluded. We developed a nomogram for estimating OS at 1, 2, and 3 years and established a corresponding risk classification system based on 1180 cases extracted from the SEER database. The nomogram is not only well discriminated and calibrated but also has strong clinical application.

## Figures and Tables

**Table 1:** Patient cohort characteristics.

Variables *n* (%)	Training cohort (*n* = 828)	Validation cohort (*n* = 352)
Age, year		
60-70	394 (47.6)	170 (48.3)
71-82	319 (38.5)	137 (38.9)
>82	115 (13.9)	45 (12.8)
Sex		
Female	324 (39.1)	145 (41.2)
Male	504 (60.9)	207 (58.8)
Race		
Black	117 (14.1)	36 (10.2)
White	656 (79.2)	301 (85.5)
Others	55 (6.6)	15 (4.3)
Marital status		
No	271 (32.7)	130 (36.9)
Yes	557 (67.3)	222 (63.1)
Primary site		
Trunk	624 (75.4)	284 (80.7)
Extremities	129 (15.6)	41 (11.6)
Facial/skull bone	75 (9.1)	27 (7.7)
Grade		
I/II	11 (1.3)	1 (0.3)
III/IV	15 (1.8)	6 (1.7)
Pre-B cell	802 (96.9)	345 (98.0)
Stage		
Localized	683 (82.5)	291 (82.7)
Distant	145 (17.5)	61 (17.3)
Surgery		
No	646 (78.0)	282 (80.1)
Yes	182 (22.0)	70 (19.9)
Chemotherapy		
No	666 (80.4)	278 (79.0)
Yes	162 (19.6)	74 (21.0)
Radiotherapy		
No	187 (22.6)	87 (24.7)
Yes	641 (77.4)	265 (75.3)

**Table 2:** Univariate and multivariate analyses of variables associated with overall survival.

Variables	Univariate analysis		Multivariate analysis	
	HR (95% CI)	*p* value	HR (95% CI)	*p* value
Age, year				
60-70	Reference		Reference	
71-82	1.926 (1.526, 2.432)	≤0.001	1.856 (1.469, 2.345)	≤0.001
>82	4.857 (3.691, 6.492)	≤0.001	4.305 (3.256, 5.692)	≤0.001
Sex				
Female	Reference			
Male	0.867 (0.707, 1.062)	0.167		
Race				
Black	Reference			
White	0.943 (0.709, 1.255)	0.689		
Other	1.269 (0.806, 1.998)	0.304		
Marital status				
No	Reference		Reference	
Yes	0.593 (0.483, 0.727)	≤0.001	0.670 (0.544, 0.824)	≤0.001
Primary site				
Trunk	Reference			
Extremities	1.117 (0.850, 1.469)	0.427		
Facial/skull bone	1.256 (0.902, 1.750)	0.177		
Grade				
I/II	Reference			
III/IV	1.616 (0.679, 3.848)	0.278		
Pre-B cell	1.048 (0.533, 2.062)	0.892		
Stage				
Localized	Reference			
Distant	1.055 (0.811, 1.372)	0.690		
Surgery		0.083		
No	Reference		Reference	
Yes	0.802 (0.624, 1.030)		0.902 (0.702, 1.160)	0.422
Chemotherapy				
No	Reference			
Yes	1.037 (0.810, 1.327)	0.774		
Radiotherapy				
No	Reference		Reference	
Yes	0.599 (0.480, 0.746)	≤0.001	0.695 (0.557, 0.868)	≤0.001

## Data Availability

The dataset from the SEER database that was generated and/or analyzed during the current study is available in the SEER dataset repository (https://seer.cancer.gov/).

## Abbreviations

AUC: Area under the curve
DCA: Decision curve analysis
EMP: Extramedullary plasmacytoma
ICD-O-3: International Classification of Diseases for Oncology
MM: Multiple myeloma
OS: Overall survival
ROC: Receiver-operating characteristic
SPB: Solitary plasmacytoma of bone
SEER: Surveillance, Epidemiology, and End Results.

## References

[1] Caers J., Paiva B., Zamagni E. (2018). Diagnosis, treatment, and response assessment in solitary plasmacytoma: updated recommendations from a European expert panel. *Journal of Hematology & Oncology*.

[2] Thumallapally N., Meshref A., Mousa M., Terjanian T. (2017). Solitary plasmacytoma: population-based analysis of survival trends and effect of various treatment modalities in the USA. *BMC Cancer*.

[3] Weberpals J., Pulte D., Jansen L. (2017). Survival of patients with lymphoplasmacytic lymphoma and solitary plasmacytoma in Germany and the United States of America in the early 21st century. *Haematologica*.

[4] Soutar R., Lucraft H., Jackson G. (2004). Guidelines on the diagnosis and management of solitary plasmacytoma of bone and solitary extramedullary plasmacytoma. *Clinical Oncology*.

[5] International Myeloma Working G (2003). Criteria for the classification of monoclonal gammopathies, multiple myeloma and related disorders: a report of the International Myeloma Working Group. *British Journal of Haematology*.

[6] Huang W., Cao D., Ma J. (2010). Solitary plasmacytoma of cervical spine. *Spine*.

[7] Baba H., Maezawa Y., Furusawa N. (1998). Solitary plasmacytoma of the spine associated with neurological complications. *Spinal Cord*.

[8] Shen X., Liu S., Wu C., Wang J., Li J., Chen L. (2021). Survival trends and prognostic factors in patients with solitary plasmacytoma of bone: a population-based study. *Cancer Medicine*.

[9] Xie L., Wang H., Jiang J. (2020). Does radiotherapy with surgery improve survival and decrease progression to multiple myeloma in patients with solitary plasmacytoma of bone of the spine?. *World Neurosurgery*.

[10] Shariat S. F., Karakiewicz P. I., Suardi N., Kattan M. W. (2008). Comparison of nomograms with other methods for predicting outcomes in prostate cancer: a critical analysis of the literature. *Clinical Cancer Research*.

[11] Cronin K. A., Ries L. A., Edwards B. K. (2014). Collaborative Staging and Its Impact on Cancer Registry Data: Information for Data Users on Analysis and Interpretation of Registry Data Preface. *Cancer*.

[12] Rousson V., Zumbrunn T. (2011). Decision curve analysis revisited: overall net benefit, relationships to ROC curve analysis, and application to case-control studies. *BMC Medical Informatics and Decision Making*.

[13] Vickers A. J., Elkin E. B. (2006). Decision curve analysis: a novel method for evaluating prediction models. *Medical Decision Making*.

[14] Dagan R., Morris C. G., Kirwan J., Mendenhall W. M. (2009). Solitary plasmacytoma. *American Journal of Clinical Oncology*.

[15] Oertel M., Elsayad K., Kroeger K. J. (2019). Impact of radiation dose on local control and survival in extramedullary head and neck plasmacytoma. *Radiation Oncology*.

[16] Ellington T. D., Henley S. J., Wilson R. J., Wu M., Richardson L. C. (2021). Trends in solitary plasmacytoma, extramedullary plasmacytoma, and plasma cell myeloma incidence and myeloma mortality by racial-ethnic group, United States 2003-2016. *Cancer Medicine*.

[17] Wang Y., Li J., Xia Y. (2013). Prognostic nomogram for intrahepatic cholangiocarcinoma after partial hepatectomy. *Journal of Clinical Oncology*.

[18] Wu S., Chen J. N., Zhang Q. W. (2018). A new metastatic lymph node classification-based survival predicting model in patients with small bowel adenocarcinoma: a derivation and validation study. *eBioMedicine*.

[19] Zhang S. L., Wang Z. M., Wang W. R., Wang X., Zhou Y. H. (2019). Novel nomograms individually predict the survival of patients with soft tissue sarcomas after surgery. *Cancer Management and Research*.

[20] Eguchi T., Bains S., Lee M. C. (2017). Impact of increasing age on cause-specific mortality and morbidity in patients with stage I non-small-cell lung cancer: a competing risks analysis. *Journal of Clinical Oncology*.

[21] Wensink M. J. (2016). Size, longevity and cancer: age structure. *Proceedings of the Biological Sciences*.

[22] Adam M. A., Thomas S., Hyslop T., Scheri R. P., Roman S. A., Sosa J. A. (2016). Exploring the relationship between patient age and cancer-specific survival in papillary thyroid cancer: rethinking current staging systems. *Journal of Clinical Oncology*.

[23] Knobel D., Zouhair A., Tsang R. W. (2006). Prognostic factors in solitary plasmacytoma of the bone: a multicenter rare cancer network study. *BMC Cancer*.

[24] Jawad M. U., Scully S. P. (2009). Skeletal plasmacytoma: progression of disease and impact of local treatment; an analysis of SEER database. *Journal of Hematology & Oncology*.

[25] Ramsingh G., Mehan P., Morgensztern D., Luo J. Q., Vij R. (2009). Prognostic factors influencing survival in solitary plasmacytoma. *British Journal of Haematology*.

[26] Dong J., Dai Q., Zhang F. (2019). The effect of marital status on endometrial cancer-related diagnosis and prognosis: a Surveillance Epidemiology and End Results database analysis. *Future Oncology*.

[27] Buja A., Lago L., Lago S., Vinelli A., Zanardo C., Baldo V. (2018). Marital status and stage of cancer at diagnosis: a systematic review. *European Journal of Cancer Care*.

[28] Li Z., Wang K., Zhang X., Wen J. (2018). Marital status and survival in patients with rectal cancer: a population-based STROBE cohort study. *Medicine*.

[29] Molloy G. J., Stamatakis E., Randall G., Hamer M. (2009). Marital status, gender and cardiovascular mortality: behavioural, psychological distress and metabolic explanations. *Social Science & Medicine*.

[30] Schone B. S., Weinick R. M. (1998). Health-related behaviors and the benefits of marriage for elderly persons. *Gerontologist*.

[31] Folk G. S., Abbondanzo S. L., Childers E. L., Foss R. D. (2006). Plasmablastic lymphoma: a clinicopathologic correlation. *Annals of Diagnostic Pathology*.

[32] Tsang R. W., Campbell B. A., Goda J. S. (2018). Radiation therapy for solitary plasmacytoma and multiple myeloma: guidelines from the international lymphoma radiation oncology group. *International Journal of Radiation Oncology • Biology • Physics*.

[33] Dimopoulos M. A., Kiamouris C., Moulopoulos L. A. (1999). Solitary plasmacytoma of bone and extramedullary plasmacytoma. *Hematology/Oncology Clinics of North America*.

[34] Mayr N. A., Wen B. C., Hussey D. H. (1990). The role of radiation therapy in the treatment of solitary plasmacytomas. *Radiotherapy and Oncology*.

[35] AvilÉS A., Huerta-GuzmÁN J., Delgado S., FernÁNdez A., Díaz-Maqueo J. C. (1996). Improved outcome in solitary bone plasmacytomata with combined therapy. *Hematological Oncology*.

[36] Kumar S., Fonseca R., Dispenzieri A. (2003). Prognostic value of angiogenesis in solitary bone plasmacytoma. *Blood*.

